# Mechanisms and Implications of Dual-Acting Methotrexate in Folate-Targeted Nanotherapeutic Delivery

**DOI:** 10.3390/ijms16011772

**Published:** 2015-01-13

**Authors:** Pamela T. Wong, Seok Ki Choi

**Affiliations:** Department of Internal Medicine, Michigan Nanotechnology Institute for Medicine and Biological Sciences, University of Michigan Medical School, Ann Arbor, MI 48109, USA; E-Mail: ptw@med.umich.edu

**Keywords:** folate, methotrexate, folate receptor, dihydrofolate reductase, PAMAM dendrimer, multivalent design, avidity, targeted delivery, nanotherapeutics

## Abstract

The rational design of a nanoplatform in drug delivery plays a crucial role in determining its targeting specificity and efficacy *in vivo*. A conventional approach relies on the surface conjugation of a nanometer-sized particle with two functionally distinct types of molecules, one as a targeting ligand, and the other as a therapeutic agent to be delivered to the diseased cell. However, an alternative simplified approach can be used, in which a single type of molecule displaying dual function as both a targeting ligand and therapeutic agent is conjugated to the nanoparticle. In this review, we evaluate the validity of this new strategy by using methotrexate, which displays multifunctional mechanisms of action. Methotrexate binds to the folate receptor, a surface biomarker frequently overexpressed in tumor cells, and also inhibits dihydrofolate reductase, an enzyme critical for cell survival and division. Thus we describe a series of fifth generation poly(amido amine) dendrimers conjugated with methotrexate, and discuss several lines of evidence supporting the efficacy of this new platform strategy based on surface plasmon resonance spectroscopy, enzyme activity assays, and cell-based studies with folate receptor (+) KB cancer cells.

## 1. Introduction

An important objective in biological nanotechnology relates to the development of nanometer-sized particles (NP) as a multifunctional delivery platform. This nanoplatform is designed for the targeted delivery of small drug molecules, therapeutic genes and imaging agents to specific cells for the treatment and detection of diseases such as cancers and other inflammatory diseases [1,2,3,4,5,6,7]. Strategies to design such delivery platforms vary in their details, but all aim to employ the NP as a carrier. Many classes of NPs have been identified for this purpose, including dendrimers such as poly(amido amine) (PAMAM) [8,9], poly(ethylene imine) (PEI) and poly(propylene imine) (PPI) [10,11,12,13], poly(lysine) [14] and poly(melamine) [15,16] as well as other NPs including poly(hydroxypropyl methacrylate) [17], poly(lactic acid-*co*-glycolic acid) (PLGA) polymers [18], carbon nanotubes [19,20] quantum dots (QDs) [21,22], iron oxide NPs (IONPs [11,23]) and transition metal (gold, platinum) [24,25,26,27] NPs.

Surface functionalization of NPs has led to further diverse types of delivery platforms that have demonstrated promising therapeutic effects and imaging capabilities in the anticancer area. Typically in this design approach, each NP is covalently conjugated with multiple molecular copies of a targeting ligand on its periphery in order to achieve multivalent tight binding [28,29,30], and further functionalized to carry therapeutic and imaging payloads for cellular delivery. Such a multivalent design plays a critical role in the cellular uptake process of the targeted NPs. It allows tight adhesion of the multivalent NP to a targeted cell surface through multivalent interactions at the interface of multiple receptor-ligand pairs. It also confers receptor-specific cell binding which is the first step towards receptor-mediated internalization of the bound NPs. This multivalent ligand strategy has been validated for targeting numerous types of tumor cells overexpressing surface biomarkers such as folic acid receptor (FAR) [31,32,33], riboflavin receptor [34,35,36], α_v_β_3_ integrin [37], prostate-specific membrane antigen (PSMA) [38], and epidermal growth factor receptor (EGFR [39]). The importance of these disease biomarkers in targeted delivery strategies is illustrated by a growing number of clinical candidates under investigation including AH 111585 [40], EC 145 (vintafolide [41,42,43]) and EC 20 (etarfolatide [41]).

Despite the rational basis supporting multivalent NP design and several existing successful proof of concept studies, several challenging issues face the therapeutic development of tumor-targeted nanotherapeutics. One of these relates to the paucity of methods to control the homogeneity and distribution of the NPs with regards to ligand density and drug load. Currently, only a few methods have been reported that allow for specialized NP functionalization with a precise number of a particular ligand molecule. These are illustrated by HPLC fractionation of PAMAM dendrimer conjugated with a hydrophobic ligand (Banaszak Holl *et al.*) [44,45,46], self-assembly of polymer units for presenting a defined number of amine aptamers (Farokhzad *et al.*) [47] and surface modification of monodisperse gold NPs with a defined number of thiol ligands (Jin *et al.*) [48]. In another approach, a ligand to drug ratio is controlled in which the PAMAM dendrimer is conjugated with a FA ligand and methotrexate (MTX), both tethered through the exact same linker (Baker Jr. *et al.*) [49]. This allows retention of a 1:1 ratio of ligand to drug molecule on each NP. Recently we (Choi and Baker Jr. *et al.*) [50,51,52] and collaborators (Banaszak Holl and Low *et al.*) [45] proposed a new strategy which is fundamentally different from the conventional approach which uses a pair of two orthogonal molecules. It is based on the use of a dual-acting single molecule that functions as both a targeting ligand to a cancer-specific receptor and as a therapeutic agent that induces cytotoxic effects following cellular internalization [45,50,51,52]. This approach requires conjugation with only a single molecule type, and thus can eliminate the introduction of more heterogeneity which occurs with the attachment of a second molecule type.

**Figure 1:**
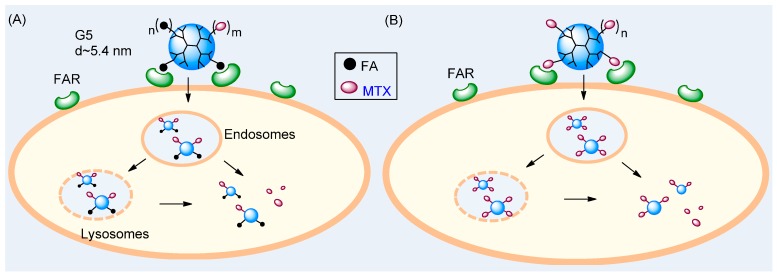
Two strategies for targeted drug delivery to a folate receptor (FAR)-overexpressing tumor cell with a fifth generation (G5) dendrimer NP. (**A**) A conventional two-molecule approach with G5(FA)_n_(MTX)_m_ presenting folic acid (FA) as a targeting ligand and carrying methotrexate (MTX) as a drug payload and (**B**) A dual-acting, single molecule approach with G5(MTX)_n_ presenting MTX as both a targeting ligand and drug payload.

In this article, we describe the mechanisms and implications of MTX as a dual-acting molecule that can both target a tumor biomarker and function as a therapeutic agent (Figure 1). MTX belongs to the class of antifolate therapeutic agents that have been used for treating cancers and rheumatoid arthritis [31,53]. Its therapeutic effect is attributed primarily to inhibition of human dihydrofolate reductase (DHFR), an enzyme localized in the cytoplasm. In addition, MTX binds to FAR because of its high structural similarity to FA. To test this MTX-based dual-acting strategy, we designed a series of multivalent dendrimer NPs, each composed of multiple MTX ligands conjugated to a fifth generation (G5) PAMAM dendrimer scaffold, and investigated their dual mechanisms of action pertinent to tumor targeted drug delivery. Firstly, we employed surface plasmon resonance (SPR) spectroscopy and determined their binding avidity to folate binding protein (FBP) immobilized to a sensor chip as a model surface for FAR(+) tumor cells. The SPR studies provide evidence supportive of multivalent tight binding of MTX-conjugated NPs. Secondly, we studied these NPs for their ability to inhibit DHFR by using a cell free-enzyme assay which shows dose-dependent blocking of the enzyme activity by the MTX molecules attached to the dendrimer. Thirdly, we determined the ability of these MTX-conjugated dendrimers to bind and kill FAR(+) KB tumor cells *in vitro*. In summary, we describe the design principle of dual-acting MTX conjugates and provide strong evidence supporting the ability of these conjugates to display the dual activities needed for an effective cancer-targeting delivery and therapeutic platform.

## 2. Results and Discussion

### 2.1. FA vs. MTX

#### 2.1.1. Binding Affinity to Folate Receptor (FAR)

FARs are expressed as three different isoforms α, β and γ [54,55]. FAR α- and β-isoform show similar FA affinity but are distinct largely in its overexpression site. For example, the FAR α-isoform is overexpressed in certain types of cancer while the FAR β-isoform is found on the surface of activated macrophages. Thus FARs are considered as attractive targets not only for cancers, but also for inflammatory arthritis [56,57] because activated macrophages are isolated in the synovial fluids from the inflamed joints of rheumatoid arthritis (RA) patients [58].

**Figure 2:**
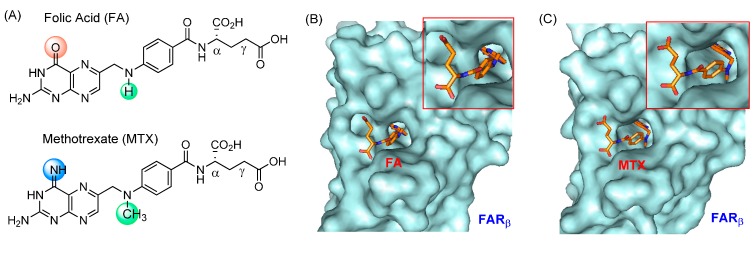
(**A**) Structures of folic acid (FA) and methotrexate (MTX); (**B**,**C**) The crystal structure of human folate receptor beta (FOLR2) bound with FA or MTX at its ligand site (PDB code 4KNO) [67]. Inset: an expanded view of each bound ligand molecule. Surface images of the receptor-ligand complexes were generated by PyMOL™ (version 1.3).

FAR_α_ is one of the biomarkers overexpressed in epithelial tumor cells in breast and ovarian cancers [59,60]. As a membrane-bound receptor, FAR_α_ plays an essential role in the cellular transport of folic acid (FA). FA has a high affinity to the FAR (*K*_D_ = 0.4 nM) [61], and its uptake occurs through FAR-mediated endocytosis. This mechanism is also used in the uptake of FA-conjugated NPs into the cytosol [62,63], and thus it plays an essential role in tumor-targeted binding and uptake of anticancer therapeutics and imaging agents carried by FA-conjugated NPs. MTX is classified as a primary member of the antifolate molecules which are characterized by their structural similarity to FA. These similarities allow them to bind with strong affinity to the receptor; however, their inhibitory effects on cell growth are opposite to the stimulatory effect displayed by FA. As shown in Figure 2A, MTX and FA are similar in their chemical structures with only two minor variations in their substitution features. As a consequence, they share similar physicochemical properties such as hydrophilicity and the prevalence of large polar surface areas (Table 1) mainly due to two carboxylic acids in glutamate that are ionized under physiological conditions (pK_a_ = 3.8, 4.8). These make FA and MTX unable to cross the cell membrane passively (low permeability constants in a Caco2 cell assay). However, unlike FA, MTX is taken up into the cell by a mechanism mediated by reduced folate carrier (RFC; *K*_D_ = 4.3 μM) [64]. In addition, the uptake of MTX is believed to occur by FAR as well because it also binds the receptor, although with an affinity (*K*_D_ = ~20–100 nM) lower than that of FA [61,65,66].

**Table 1:** Summary of physicochemical properties and pharmacology of folic acid (FA) and methotrexate (MTX).

	*M*w (g/mol)	*^a^*^,*b*^ clogD	*^b^*^,*c*^** tPSA (Å^2^)	*^d^* Permeability (×10^−6^ cm/s)	*K*_D_ (Receptor)	*K*_i_ (Cellular Target)
FA	441.4	−5.82	208	1.7 [68]	0.4 nM (FAR) [61]	0.48 μM (DHFR) [69]
MTX	454.4	−4.98	211	1.2 [68]	20–100 nM (FAR) [61,65,66]; 4.3 μM (RFC) [64]	1.2 nM (DHFR) [70]; 13 μM (TYMS) [71]; 96 μM (dCK) [72]

*^a^* D = distribution coefficient = [Drug]_octanol_/[Drug]_buffer, pH 7.0_; *^b^* Calculated by using ACD/Labs Software 11.02; *^c^* tPSA = total polar surface area; *^d^* Caco2 assay; abbreviations: FAR = folate receptor, RFC = reduced folate carrier, DHFR = dihydrofolate reductase, TYMS = thymidylate synthase, dCK = deoxycytidine kinase.

Very recently, X-ray crystal structures have been determined at a high resolution for a FAR protein in complex with FA [67,73] or MTX [67]. Each structure shows that either ligand molecule binds into the receptor pocket with an almost identical orientation and geometry (Figure 2B,C). In the structure, a pteridine residue in the bound molecule is positioned deep in the pocket while the two carboxylate (α, γ) groups of the L-Glu residue stick out near the entrance of the pocket, making each of them useful for FAR targeting by covalent conjugation to a NP. Although MTX has a lower FAR affinity, its use as a ligand is still effective for FAR targeting if a multivalent design approach [28,29,30] is applied which can offer very tight binding compared to a weak monovalent binding interaction.

#### 2.1.2. Enzyme Pharmacology

MTX is a therapeutic agent important for the treatment of various cancers and inflammatory arthritis [74,75]. Its therapeutic activity is attributed to its ability to inhibit metabolic processes in the cytoplasm. It shows a potent inhibitory activity against human dihydrofolate reductase (DHFR), a cytosolic enzyme that catalyzes the reduction of dihydrofolate to tetrahydrofolate, and thus plays an essential role in purine biosynthesis. Blocking this catalytic process with MTX (*K*_i_ = 1.2 nM) [70] leads to the inhibition of cell proliferation and growth, and consequently cytotoxicity. However, a part of the cytotoxic activity of MTX is contributed by its inhibition of other enzyme targets including thymidylate synthase (TYMS; *K*_i_ = 13 μM) [71] and deoxycytidine kinase (dCK; *K*_i_ = 96 μM) [72], each though with a lower potency than DHFR.

### 2.2. Binding to a FAR(+) Model Surface as Studied by SPR Spectroscopy

**Figure 3:**
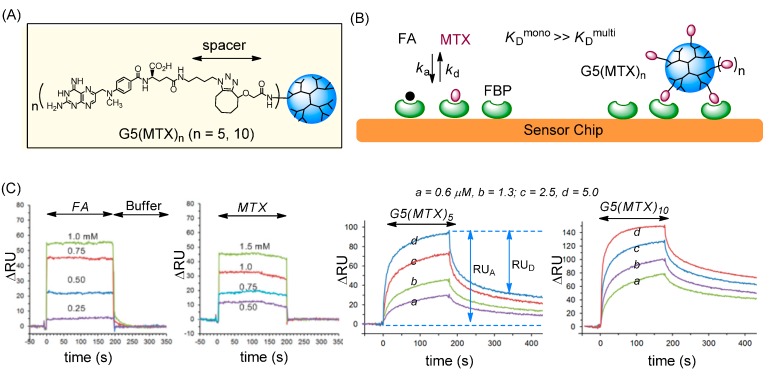
(**A**) Structure of G5(MTX)_n_ (*n* = 5, 10) dendrimer conjugated with MTX via cyclooctyne-azide click chemistry; (**B**) Schematic for binding of FA, MTX and G5(MTX)_n_ to the surface of a folate binding protein (FBP)-immobilized CM5 sensor chip; (**C**) Overlaid dose-dependent SPR sensorgrams [51].

As noted earlier, MTX binds to FAR with a lower affinity than FA. However, we proposed that its suboptimal affinity could be overcome by using a multivalent ligand configuration that allows tight adsorption to a FAR-overexpressing cell surface (Figure 3). We employed SPR spectroscopy to validate this design for FAR targeting by multivalent MTX ligands. For the SPR experiments, we prepared a model FAR surface by immobilization of bovine folate binding protein (FBP) to a CM5 sensor chip at a surface density of 3 × 10^11^ FBP per mm^2^. This receptor density is comparable to the overexpression level of FAR in ovarian and endometrial cancers in which the receptor is expressed at levels 10–20-fold higher than normal epithelial cells [76,77].

#### 2.2.1. Monovalent Ligands

SPR sensorgrams were acquired with monovalent ligands (FA, MTX) as shown in Figure 3 [50,51]. The sensorgrams for each of these ligands were analyzed and fit to a monovalent Langmuir binding model. The kinetic rate constants (*k*_a_, *k*_d_) were extracted as summarized in Table 2. The results show that MTX binds to the FBP receptor with micromolar range dissociation constants (*K*_D_ = 2.4–4.0 × 10^−5^ M), corresponding to an affinity of ~2- to 20-fold lower than that of FA (*K*_D_ = 2.0–11 × 10^−6^ M). This is due primarily to the higher association rate (*k*_a_) of FA (1.1 × 10^3^ s^−1^·M^−1^) than MTX (7.0 × 10^2^ s^−1^·M^−1^) [50] since their dissociation rates (*k*_d_) are similar (Table 2).

**Table 2:** Kinetic and equilibrium dissociation constants of FA, MTX and MTX-conjugated dendrimers G5(MTX)_n_ to folate binding protein (FBP) immobilized onto a CM5 sensor chip. *^a^*

	FA [50,51]	MTX [50,51]	G5(MTX)_n_ (*n* = 5) [51]	G5(MTX)_n_ (*n* = 10) [51]
*^b^* *k*_d_ (s^−1^)	1.2 × 10^−2^	1.7 × 10^−2^	2.0 × 10^−4^	7.8 × 10^−5^
*^c^* *K*_D_ (M)	2.0–11 × 10^−6^	2.4–4.0 × 10^−5^	2.1 × 10^−9^	5.5 × 10^−10^
*^d^* β	-	1	19,045 (3810)	72,727 (7273)

*^a^* receptor density = 3 × 10^11^ FBP molecules/mm^2^; *^b^* *k*_d_ = dissociation rate constant; *k*_a_ = association rate constant; *^c^* *K*_D_ = equilibrium dissociation constant = *k*_d_/*k*_a_; *^d^* β = multivalent binding enhancement = *K*_D_^mono^/*K*_D_^multi^ (parenthesis = valency (n) corrected = β/n).

#### 2.2.2. Multivalent MTX Ligands

We then studied the effect of multivalent association between multiple FBP receptors present on the surface and a dendrimer conjugated with multiple MTX molecules G5(MTX)_n_ (*n* = 5, 10). Each of these G5(MTX)_n_ conjugates were synthesized by copper-free azide-alkyne click chemistry which was achieved by incubation of an azide-terminated MTX molecule with a cyclooctyne-attached G5 dendrimer [52]. SPR binding studies were performed for each of the dendrimers (Figure 3C) and their binding kinetics were measured. Each dendrimer-MTX conjugate bound effectively to the FBP surface even at submicromolar doses as low as 0.1 μM at which binding of free FA or MTX is not detectable. Dendrimer binding was highly FBP specific, as the binding signal on the FBP surface (flow cell 1) was high, with relatively no binding observed on the non-FBP reference surface (flow cell 2). In contrast, G5(MTX)_0_, a dendrimer control not clicked with MTX, failed to show any adsorption to either channel of an otherwise identical sensor chip. Lastly, G5(MTX)_10_ with a higher MTX valency showed greater adsorption (RU_A_) and lower RU_D_ (slower dissociation) than G5(MTX)_5_. This difference is indicative of a positive correlation between MTX valency (n) and avidity.

We next determined the kinetic rate and equilibrium dissociation constants for G5(MTX)_n_ by nonlinear regression analysis as summarized in Table 2. Each multivalent dendrimer had an extremely slow dissociation rate (*k*_d_ = 7.8–20 × 10^−5^ s^−1^) in contrast to the free FA or MTX molecules which have rapid dissociation rates (*k*_d_ = 1.2–1.7 × 10^−2^ s^−1^). Such slow dissociation is a hallmark of tight multivalent interaction as reported in numerous other multivalent systems [28,29,30]. The *K*_D_ values determined for G5(MTX)_5_ and G5(MTX)_10_ are 2.1 × 10^−9^ and 0.55 × 10^−9^ M, respectively. These values reflect a remarkable enhancement of binding avidity by a factor of ~19,000 to ~73,000 (β = [*K*_D_^mono^/*K*_D_^multi^]) relative to free monovalent MTX. In summary, the SPR study demonstrates that multivalent dendrimers conjugated with MTX binds selectively to the FAR model surface and much more tightly than free MTX. It strongly supports that MTX can serve as an efficient ligand for FAR targeting in lieu of FA.

### 2.3. Dendrimer Binding to FAR(+) Cells in Vitro Studied by Fluorescence Confocal Microscopy and Flow Cytometry

In order to determine whether dendrimer binding to the model surface reflected the ability of the dendrimer to associate with live cells, we performed binding studies of MTX-conjugated dendrimers with tumor cells that express a high level of FAR. Dendrimer conjugates studied here included (FITC)G5(MTX)_n_ (*n* = 5, 7.5) [50,78] and (TAMRA)G5(MTX)_n_ (*n* = 10) [52], each fluorescently labeled but presenting otherwise MTX ligand alone.

First, fluorescein isothiocyanate (FITC)-labeled dendrimers (FITC)G5(MTX)_n_ (*n* = 5, 7.5) were synthesized by covalent conjugation of glutaric acid (GA) modified dendrimer G5(GA) with a MTX derivative made through the attachment of an amine-terminated linker at L-Glu. Each dendrimer bound to FAR(+) KB cells in a dose-dependent manner at concentrations up to 1 μM while the dendrimer with a higher MTX valency (*n* = 7.5) showed a slightly greater level of cellular binding and uptake [50]. Interestingly, each dendrimer did not show a dose-dependent saturation binding curve at the high concentration range which is often displayed by FA-conjugated dendrimers [79]. This lack of binding saturation might be attributable to a number of potential differences between FA and MTX such as lower binding avidity and slower rate of cellular uptake by MTX. However, like FA-conjugated dendrimers, MTX-conjugated dendrimer bound specifically to FAR since its binding could be blocked by co-incubation with free FA, though only when added at a high concentration (50 μM). The uptake of (FITC)G5(MTX)_n_ (*n* = 5, 7.5) in KB cells was also confirmed by confocal microscopy. Intense FITC-related green fluorescence was observed in the cytoplasm, indicating that the dendrimer particles were internalized. These cell-binding studies suggest that FAR molecules are the surface receptor targeted by these MTX conjugates and are involved in the internalization of the conjugate.

**Figure 4:**
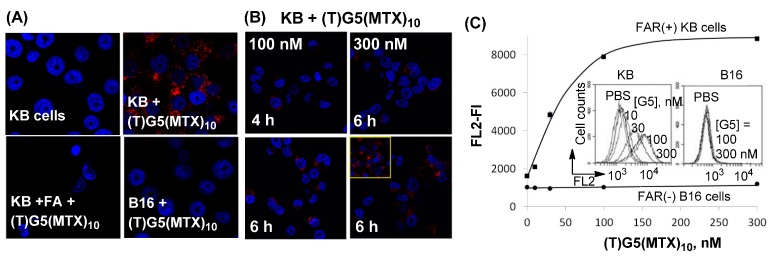
(**A**,**B**) Confocal microscopy images showing FAR-specific and time-dependent binding and uptake of (TAMRA)G5(MTX)_10_ in FAR(+) KB and FAR(−) B16-F10 cells [51,52]; (**C**) Flow cytometric analysis. Inset: histograms showing the FL2 fluorescence (FL) of 10,000 KB or B16 cells. TAMRA = 5-carboxytetramethylrhodamine. Staining: Nuclei (DAPI; blue); Cytosol (TAMRA; red).

Second, in addition to FITC-labeled dendrimer, TAMRA-labeled dendrimer (TAMRA)G5(MTX)_10_ which is more fluorescently intense was also designed to determine its FAR-specific binding in FAR(+) tumor cells. Its binding and uptake features were studied by confocal microscopy and flow cytometry in two cell lines with and without FAR: FAR(+) KB and FAR(−) B16-F10 cells [51,52]. The red fluorescence corresponding to TAMRA was detected in the cytoplasm of FAR(+) KB cells, indicating that the dendrimer NPs are bound and internalized by KB cancer cells (Figure 4). In order to determine FAR specificity, dendrimer binding was also performed with co-incubation with free FA (Figure 4C). Under these competitive ligand conditions, dendrimer binding and uptake was not detectable, suggesting that its cell binding is mediated by a FAR-specific mechanism. Finally, the dendrimer failed to bind FAR(−) B16-F10 cells in the absence of FA, further confirming the FAR specificity of the dendrimer-cell interaction. Flow cytometry was employed for quantitative analysis of the cellular binding of (TAMRA)G5(MTX)_10_ (Figure 4E). This dendrimer bound to FAR(+) KB cells in a dose-dependent manner, but did not bind FAR(−) B16 cells. The results of the confocal and flow data acquired with (TAMRA)G5(MTX)_10_ provide strong evidence supportive of its FAR-targeted cellular binding and uptake.

### 2.4. Inhibition of Dihydrofolate Reductase in a Cell-Free Condition

**Figure 5:**
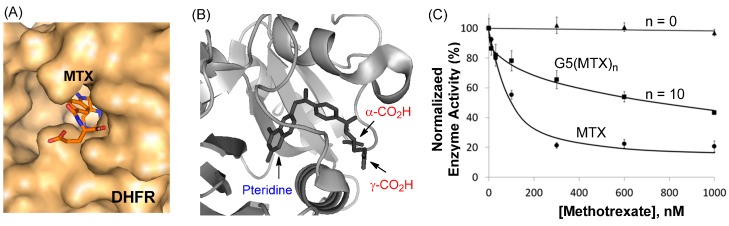
(**A**,**B**) A crystal structure of a MTX molecule in complex with dihydrofolate reductase (DHFR) at its catalytic site (PDB code 1DDS [80], 1U72 [81]). The protein (surface, cartoon) and MTX molecule (stick) model of the enzyme-drug complex was generated by PyMOL™ (version 1.3); (**C**) Inhibition of human DHFR enzyme activity by MTX and G5(MTX)_n_ (*n* = 0, 10; Figure 3) conducted in a standard enzyme assay [52]. The concentrations on the X-axis are expressed as MTX or MTX equivalents rather than dendrimer.

A number of X-ray crystal structures have been solved for the complexes of DHFR with MTX [80,81]. Like in the complex of FAR with MTX as discussed above, each DHFR complex shows the pteridine head group (hidden) bound deep into the pocket, while the L-Glu carboxylic acids from the drug molecule are anchored near the entrance to the enzyme catalytic pocket (Figure 5A,B). Thus, it is expected that conjugation of MTX to the dendrimer through attachment at either carboxylate residue would allow retention of the enzyme binding, and consequently, inhibitory activity of the tethered MTX molecule.

In order to evaluate the ability of the dendrimer-MTX conjugates to inhibit DHFR after cellular entry, an enzyme activity assay was performed using recombinant human DHFR, which is the primary cytosolic enzyme target inhibited by MTX. The inhibition assay requires only a short period of incubation (≤5 min) in a neutral solution (PBS, pH 7.4). During this short assay time, the amide linkage that tethers the MTX molecule to the dendrimer remains completely stable and no free MTX is released.

MTX-conjugated dendrimers, G5(MTX)_n_ which were tested in this assay comprise a diverse group of conjugates, each containing MTX molecules, and attached through an amide linker at its L-Glu residue, with variation in drug number (n) [50,52,78]. First, G5(MTX)_10_ (Figure 3) inhibited DHFR activity in a dose-dependent manner with an IC_50_ value of approximately 1000 nM as shown in Figure 5C. However, this MTX conjugate was less potent than free MTX (IC_50_ ≈ 100 nM). We believe that this reduced activity might be related to the lower affinity of the conjugated MTX than free MTX to DHFR possibly due to the unfavorable steric repulsion at the interface between the enzyme and the dendrimer. Such enzyme inhibition is specific to MTX since G5(MTX)_0_, a control dendrimer that lacks MTX, showed no inhibition at doses as high as 1000 nM.

The assay was then performed for dendrimers conjugated with MTX by using a variable linker length from long (3–4 nm) to no spacer. Two MTX conjugates, G5(MTX)_n_ (*n* = 4, 12) [78], each tethered through a long spacer (3–4 nm), inhibited the enzyme activity with IC_50_ values of ≥800 nM. However, this enzyme inhibition is approximately 8-fold less potent than free MTX (IC_50_ ≈ 100 nM). In contrast, other conjugates, G5(MTX)_n_ (*n* = 3, 5) [78] where each MTX is conjugated with either a shorter (<2 nm) or no spacer, showed only weak activity (<40% inhibition at 1000 nM).

In summary, the DHFR assay results demonstrated that MTX-conjugated dendrimer is able to inhibit DHFR through the tethered MTX. However the efficiency of the enzyme inhibition is lowered when MTX is tethered to the dendrimer as compared to free MTX. Furthermore, the linker length plays an important role in influencing the inhibition activity, and needs to meet a certain threshold distance and linker flexibility in order to allow for the tethered MTX to bind in the enzyme catalytic pocket.

### 2.5. In Vitro Cytotoxicity Studied by XTT Assay

**Figure 6:**
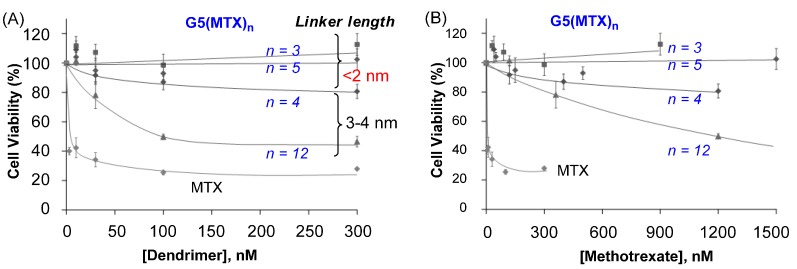
*In vitro* cytotoxicity of G5(MTX)_n_ in FAR(+) KB cells determined by an XTT assay [78]. Each data point represents a mean value (±SD). Doses for each conjugate on the *X*-axis are expressed as either dendrimer (**A**) or MTX (**B**) concentration.

We investigated whether G5(MTX)_n_ dendrimers are cytotoxic to FAR(+) KB cells by using an XTT (sodium 3'-[1-[(phenylamino)-carbony]-3,4-tetrazolium]-bis(4-methoxy-6-nitro)benzene-sulfonic acid hydrate) assay. First, MTX conjugates linked through variable spacer lengths (<2 nm; 3–4 nm) were determined as shown in Figure 6 [50]. G5(MTX)_12_, the conjugate with a high number of MTX molecules attached through a long spacer (3–4 nm), induced dose-dependent cytotoxicity (IC_50_ ≈ 100 nM). While it is less potent than free MTX (IC_50_ ≤ 10 nM), this conjugate is significantly more active than other conjugates carrying MTX tethered through a shorter spacer G5(MTX)_n_ (*n* = 3, 5; spacer < 2 nm) which failed to induce any significant cytotoxicity. Negative control dendrimers such as fully acetylated dendrimer or other dendrimers without MTX conjugated did not show any cytotoxicity when tested under an identical condition. This lack of activity indicates that the MTX payload is responsible for killing tumor cells. The results observed in this XTT assay are in good agreement with the conclusion derived above in inhibition of DHFR activity in a cell-free solution.

It is notable that G5(MTX)_4_, the conjugate having a lower number of MTX, though attached through the same long spacer (3–4 nm), showed only a modest induction of cytotoxicity. We believe that this reduced cytotoxicity compared to G5(MTX)_12_ is possibly due to other factors, primarily, its lower avidity in FAR binding as suggested by the SPR study. This notion that the binding avidity relates to the efficiency of cytotoxicity is also supported by other conjugate series G5(MTX)_n_ (*n* = 5, 10) with MTX tethered with a long cyclooctyne linker (Figure 3). G5(MTX)_10_, which binds to FBP receptor ~4-fold more tightly than G5(MTX)_5_ (Table 2), was more cytotoxic to KB cells than G5(MTX)_5_ (not shown). It showed approximately 50% inhibition of cell viability at 30 nM (IC_50_) while the G5(MTX)_5_ showed only 10% inhibition at the same dose with an IC_50_ value of ≥300 nM. Thus, the higher cytotoxicity is likely attributable to its greater avidity as suggested by the SPR binding study.

The DHFR assay and the cytotoxicity study as discussed show that free MTX has greater activity than MTX-conjugated dendrimers. However, despite its more potent activity *in vitro*, MTX enters cells through an uptake process facilitated by FARs as well as by reduced folate carrier (RFC), which is ubiquitously expressed in all cell types. Involvement of such a ubiquitous mechanism in its cellular uptake causes the well known broad range of side effects of MTX. In contrast, dendrimer conjugates are not internalized by a passive membrane diffusion mechanism or by the RFC route. Rather it is taken up by the FAR-mediated mechanism which provides specificity for targeting certain tumor cells with upregulated FAR. Therefore the dendrimer conjugates show almost no or much weaker cytotoxicity in FAR(−) B16 cells than free MTX [52]. Given this difference in the mechanisms of cellular uptake, we believe that MTX-conjugated dendrimers will display higher efficacy with fewer adverse effects *in vivo* than free MTX [31]. In addition, conjugation of MTX to the dendrimer brings other benefits such as in drug pharmacokinetics by extending its half-life in the blood over free MTX as well-demonstrated in polymer-based pharmaceutics [31,82].

### 2.6. Role of MTX Release in Vitro

As discussed above, our studies demonstrate that MTX-conjugated dendrimers are taken up by a FAR(+) tumor cell and kill the cell. The DHFR assay conducted in a cell-free solution suggests that the tethered MTX molecule could inhibit the enzyme catalytic action if its spacer is sufficiently long and flexible. While these results give important clues on the active species and mechanism of action after cellular entry, the exact nature and species of the MTX payload responsible for the cytotoxicity is poorly understood.

In order to define the role or need of MTX release in determining its cytotoxic action in the cell, we designed several control studies. First, we investigated a FA-conjugated dendrimer system G5(FA)_4_(MTX)_5_ in which MTX is attached to the dendrimer at its L-Glu residue via an ester linkage instead of a stable amide once used for those conjugates described above [83]. This conjugate inhibited human DHFR in the enzyme assay and was highly cytotoxic in KB cells. We investigated its chemical stability at low pH (≤5), a condition that mimics the acidic environment of endosomes (pH ≈ 5–6.5) which contain conjugates that have been taken up via FAR-mediated endocytosis. Interestingly, the ester linkage was resistant to hydrolysis, and the conjugate failed to release the MTX payload, suggesting no significant role of MTX release in the observed cytotoxicity.

**Figure 7:**
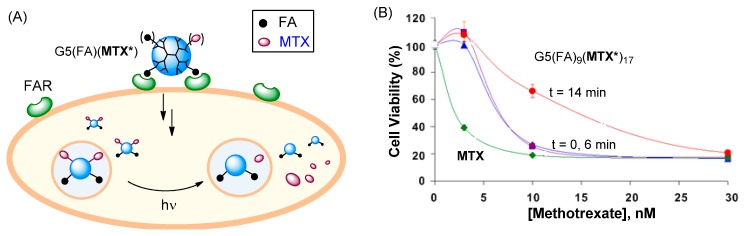
(**A**) Proposed schematic illustrating the concept of light-triggered MTX release; (**B**) *In vitro* cytotoxicity of G5(FA)_9_(MTX*)_17_ in FAR(+) KB cells, before (control; *t* = 0) and after UV irradiation (*t* = 6 or 14 min) [84]. MTX* = MTX linked with a photocleavable linker.

Second, we developed a novel strategy that would enable precise control of drug release in the cell [84,85]. In this strategy, a dendrimer, G5(FA_9_)(MTX*)_17_ which contains MTX attached with a photocleavable ortho-nitrobenzyl (ONB) linker that can be cleaved by exposure to UV light was used [84]. This allows controlled release of MTX molecules from the dendrimer by using a light trigger. This dendrimer was evaluated in FAR(+) KB cells for its cytotoxicity before and after UV light exposure. As summarized in Figure 7, each treatment condition showed dose-dependent cytotoxicity. Without light exposure (*t* = 0 min, control), a maximal 80% decrease in cell viability was observed relative to untreated cells. This potency is equivalent to an IC_50_ value of ~7 nM, indicating that this conjugate is approximately three-fold less potent than free MTX. Cytotoxicity measured following a 6 min exposure led to almost no change in the inhibition compared to no UV conditions. In a separate release study analyzed by HPLC, 6 min exposure was found to be sufficient for full MTX release, suggesting that the cytotoxicity before and after MTX release is almost identical [84]. However, a prolonged exposure (*t* = 14 min) decreased the activity (IC_50_ ≈ 15 nM) possibly as a result of partial degradation of MTX molecules, as suggested by HPLC analysis. In summary, the light-controlled drug release shows that the targeted delivery of active MTX can be achieved effectively with or without drug release.

## 3. Conclusions

As a potent inhibitor of DHFR, MTX serves as an important therapeutic agent for the treatment of various types of cancers and inflammatory diseases. However, due to its dose-limiting adverse effects, MTX has been a subject of active investigation in targeted drug delivery. A conventional approach investigated for its delivery to FAR-overexpressing tumor cells uses a nanocarrier conjugated with FA as a targeting ligand. However, MTX plays another role as a targeting ligand to FAR, and its dual activity offers a rare opportunity to develop an alternative, simplified platform for drug delivery.

In this review, we discussed various aspects in the design and functional activity of this MTX-based nanoplatform, and provided multiple lines of evidence supporting its validity and practicality. We have performed proof of concept studies with numerous types of multivalent MTX-conjugated dendrimers, each designed with variation in MTX valency, linker length and functionality. First, these multivalent MTX dendrimers bind to a FAR model surface three to four orders of magnitude more tightly than free MTX or FA as determined by SPR. Such high avidity observed in the model surface is consistent with its specific binding and uptake by FAR(+) tumor cells *in vitro*; Second, conjugation of MTX to the dendrimer allows retention of the ability of the attached MTX to inhibit a human DHFR enzyme as potently as free MTX; Third, this enzyme inhibition activity is translatable *in vitro* in cell studies, as these dendrimers were potently cytotoxic to FAR(+) KB cells. It is notable that optimal activity of this dual-functional platform is determined primarily by certain design factors such as MTX valency and linker length. Collectively, these studies provide strong evidence supporting the validity and efficacy of a tumor targeting nanodelivery strategy designed with dual-acting MTX.

We believe that this MTX-based dual-acting strategy has broad implications in targeted delivery. First, MTX-conjugated dendrimers have potential as a therapeutic approach for multivalent targeting and inhibition of FAR-overexpressing diseased cells such as cancer cells and activated macrophages [55,58] which are one of the causative agents for rheumatoid arthritis (RA) [31,32,56,86]; Second, this concept is similarly applicable for other drug molecules. A recent study [67] reveals that FAR binds two other antifolate molecules with nanomolar affinity constants including aminopterin (*K*_D_ = 60–144 nM) and pemetrexed (4.5–54 nM), an anticancer agent approved for treating a lung cancer. Thus like MTX, each of these agents has a FAR-targeting and therapeutic function that can be developed for FAR-targeted delivery; Third, we believe that this dual-acting dendrimer NP can be further applied for drug complexation for combination therapy. We have demonstrated that dendrimer NPs serve as an efficient delivery vehicle due to their ability to carry genes [34] and small drug payloads [86,87] by a mechanism of non-covalent complexation. Thus, this dual-acting NP has ability to carry a second therapeutic agent for co-delivery to FAR(+) cancer cells. We expect these topics constitute the subject of future studies.
